# Immunogenicity of rat-neu^+^ mouse mammary tumours determines the T cell-dependent therapeutic efficacy of anti-neu monoclonal antibody treatment

**DOI:** 10.1038/s41598-020-60893-8

**Published:** 2020-03-03

**Authors:** Heng Sheng Sow, Hreinn Benonisson, Conny Brouwers, Margot M. Linssen, Marcel Camps, Cor Breukel, Jill Claassens, Thorbald van Hall, Ferry Ossendorp, Marieke F. Fransen, J. Sjef Verbeek

**Affiliations:** 1 Department of Human Genetics, Leiden University Medical Centre, Leiden, The Netherlands; 2 Department of Immunohematology and Blood Transfusion, Leiden University Medical Centre, Leiden, The Netherlands; 3 Department of Medical Oncology, Leiden University Medical Centre, Leiden, The Netherlands; 4 Present Address: Department of Pulmonary Diseases, Amsterdam University Medical Centre, Amsterdam, The Netherlands

**Keywords:** Breast cancer, Immunotherapy

## Abstract

The use of Trastuzumab (Herceptin), a monoclonal antibody (mAb) targeting HER2/neu, results in an increased median survival in Her2^+^ breast cancer patients. The tumour mutational burden and the presence of tumour infiltrating lymphocytes (TILs) clearly correlate with response to trastuzumab. Here, we investigated if the immunogenicity of the transplantable rat-neu^+^ tumour cell line (TUBO) derived from a BALB/c-NeuT primary tumour is associated with the response to anti-neu mAb therapy. We compared the TUBO tumour outgrowth and tumour infiltrating T cells in isogenic (BALB/c-NeuT) and non-isogenic (WT BALB/c) recipient mice. Furthermore, therapeutic efficacy of anti-neu mAb and the contribution of T cells were examined in both mouse strains. The outgrowth of untreated tumours was significantly better in BALB/c-NeuT than WT BALB/c mice. Moreover, tumour infiltrating T cells were more abundantly present in WT BALB/c than BALB/c-NeuT mice, showing that the TUBO tumour was more immunogenic in WT BALB/c mice. In TUBO tumour bearing WT BALB/c mice, anti-neu mAb therapy resulted in an increase of tumour infiltrating T cells and long-term survival. When T cells were depleted, this strong anti-tumour effect was reduced to an outgrowth delay. In contrast, in TUBO tumour bearing BALB/c-NeuT mice, treatment with anti-neu mAb resulted only in tumour outgrowth delay, both in the presence and absence of T cells. We concluded that in immunogenic tumours the response to anti-neu mAb therapy is enhanced by additional T cell involvement compared to the response to anti-neu mAb in non-immunogenic tumours.

## Introduction

Overexpression of oncogenic Her2 protein occurs in 15–20% of breast cancers and is associated with highly aggressive disease. Trastuzumab, a humanized IgG1 monoclonal antibody targeting Her2, is the standard therapy for Human epidermal growth factor receptor 2 (HER2/Erbb2/neu) overexpressing breast cancer owing to its apparent efficacy in adjuvant and neoadjuvant uses^1,2^. This antibody was designed to disrupt the ligand-independent HER2-HER2 interaction resulting in rapid inhibition of pro-survival signalling pathways, leading to cell cycle arrest of the cancer cells^3,4^. The clinical success of Trastuzumab has paved the way for devising novel Her2 targeting approaches in breast cancer treatment. To date, two other therapeutic agents are available to inhibit her2 mediated signalling, a monoclonal antibody (pertuzumab) and tyrosine kinase inhibitors (lapatinib, neratinib). However, a recent clinical study has shown that Trastuzumab therapy increases the pathological complete response (pCR) in patients more than laptinib^5^. This can be attributed to the ability of Trastuzumab to engage the immune system to achieve tumor killing. Several preclinical studies have suggested that innate immune responses are essential to anti-Her2/neu mAb cancer therapies through the recruitment of Fcγ receptor (FcγR) expressing immune cells which can mediate antibody-dependent cellular cytotoxicity (ADCC)^6–8^. This is supported by the observation that in patients the efficacy of Trastuzumab correlates positively with the presence of allelic variants of FcγRIII with higher affinity for IgG^9,10^.

In addition, *in vivo* preclinical studies have demonstrated adaptive immune responses being also essential for the therapeutic efficacy of anti-Her2/neu mAb^8,11^. These studies were mostly performed using a transplantable mammary tumor derived from a spontaneous primary tumor of an inbred BALB/c-NeuT transgenic female mouse (H-2^d^)^12^. Females of these transgenic animals, which are hemizygous for the constitutively activated/mutated rat-Her2 gene (NeuT) under control of the MMTV promoter, develop invasive mammary carcinomas in all ten mammary glands^13^. This model recapitulates the anatomical location and pathophysiology observed in human Her2^+^ breast cancer, thus allowing the evaluation of potential cancer immunotherapies. A transplantable cell line derived from a spontaneous rat-neu^+^ mammary tumour has been preferably used in many laboratories for cancer immunotherapy studies, on the basis of a short-latency periods and reproducibility. However, instead of transplanting these cells into syngeneic BALB/c-NeuT mice, WT BALB/c mice are often used as the recipient of the tumour cells in the majority of the studies. Using such a transplantable tumor cell line, called TUBO, and WT BALB/c and F1 BALB/c FVB/N-Tg (MMTV-neu) mice as a recipient, findings of Park^8^ and Mortenson^14^ *et al*. suggest that adaptive immunity, in particular the role of CD4^+^ and CD8^+^ T cells, is essential for the anti-neu mAb-mediated tumour regression. However, these results raise some concerns. As TUBO cells express activated/mutated rat-neu being a foreign antigen in WT BALB/c, this tumour cells potentially triggers anti-rat-neu adaptive immunity which contributes to anti-neu mAb therapy. Therefore, we studied the role of the adaptive T cell immunity in full syngeneic setting by transplanting TUBO cells on BALB/c-NeuT mice. Here, we show an association between tumour immunogenicity and the therapeutic efficacy of anti-neu mAb depending on antigenic differences between the tumor and the recipient mouse strain. We explored whether and to what extent both CD8^+^ and CD4^+^ T cells are involved in the therapeutic effects of anti-neu mAb in both WT BALB/c and BALB/c-NeuT tumor bearing mice.

## Materials and Methods

### Mice

WT BALB/c female mice were purchased from Charles River (L’Arbresle, France). MMTV BALB/c-NeuT transgenic^12,13,15^ mice expressing activated rat-neu under the control of MMTV promoter were kindly provided by Karin de Visser (NKI, Amsterdam) and maintained by mating BALB/c-NeuT males with WT BALB/c females. The mice were housed in the SPF animal facilities of the Central Animal Facility (PDC) of the Leiden University Medical Center (LUMC). BALB/c-NeuT transgenic mice were bred in house and routinely checked for their genotype by PCR. All mice were housed in individually-ventilated-cage (IVC) systems under specific pathogen-free conditions and used at 6–12 weeks of age. The health status of the animals was monitored over time. Animals tested negative for all agents listed in the FELASA (Federation of European Laboratory Animal Science Associations) guidelines for SPF mouse colonies^16^. All mouse studies were approved by the Central Committee for Animal Research (Centrale Commissie Dierproeven, CCD) and Animal Welfare Body (AWB) (Instantie voor Dierenwelzijn, IvD). Experiments were performed in accordance with the Dutch Act on Animal Experimentation and EU Directive 2010/63/EU (‘On the protection of animals used for scientific purposes’).

### Antibodies

Anti-neu mAb (clone 7.16.4, mIgG2a) hybridoma^17^ was kindly provided by Mark I. Greene, University of Pennsylvania. Anti-neu mAb, anti-CD8 mAb (clone 2.43)^18^ and anti-CD4 mAb (GK 1.5)^19^ were produced and purified at Leiden University Medical Center (LUMC).

### Tumour models and treatment

The TUBO tumour cell line^12^ (kindly provided by Pier-Luigi Lollini, University of Bologna) was cultured in Iscove’s modified Dulbecco’s medium (IMDM) (Lonza) Supplemented with 16% Fetal Calf Serum (FCS) (Greiner), 25 µM 2-mercaptoethanol and 100 IU/ml penicillin/streptomycin (Gibco). Cell lines were mycoplasma and MAP-tested before the start of experiments. TUBO Tumour cells (5 × 10^5^) were injected subcutaneously in 200 µl of PBS on the right flank of 6–12 weeks old BALB/c-NeuT or WT BALB/c female mice. TUBO bearing WT BALB/c or BALB/c-NeuT mice were treated with three intraperitoneal injections of 100 µg of anti-neu mAb (clone 7.16.4) at day 10, 15 and 20. Tumours were measured 3 times per week with a calliper and the size was calculated by multiplying the tumour diameters in two dimensions. Mice were sacrificed when established tumours reached a size of 100 mm^2^.

For the anti-neu mAb treatment efficacy studies using BALB/c-neuT female mice^12^, the mammary glands of BALB/c-neuT mice were inspected weekly for tumour appearance from the twelfth week of age. When a cumulative spontaneous tumour burden of 20 mm^2^ was reached, the mice were treated with three intraperitoneal injections of 100 µg of anti-neu mAb (clone 7.16.4) at day 10, 15 and 20. The spontaneous tumours were measured 3 times per week with a calliper and their size was calculated by multiplying the tumour diameters in two dimensions. Cumulative tumour burden was calculated as the sum of all individual tumour sizes. Mice were sacrificed when established spontaneous tumours reached a cumulative tumour burden of 15 × 15 mm.

### Flow cytometry

Flow cytometry analysis for tumour infiltrating CD45^+^, CD4^+^ and CD8^+^ were performed as previously described^20^. In brief, tumours were harvested into 1 mL of non-supplemented IMDM media in 24-well plates and manually dissociated into small pieces with scalpels, incubated with 2.5 mg/mL Liberase TL (Roche) for 20 minutes at 37 °C and single-cell suspensions were made using 70-µm cell strainers (BD Biosciences). FcγRs were blocked with 10% normal mouse serum and anti-mouse CD16/CD32 antibody (2.4G2). Cell surface staining was performed using the following antibodies: anti-mouse CD8α (clone 53-6.7), CD4 (clone L3T4), CD3ε (clone 145-2c11)/ TCRβ chain (clone H57–597), CD45.2 (clone 104). Dead cells were excluded based on 7-AAD (Invitrogen). Analysis were performed using LSRII cytometer (BD) using FacsDIVA software (BD) and FlowJo Software (Tree Star).

### Statistical analyses

Data was analysed using Prism 7.0 (GraphPad Software). Statistical significance was calculated using the Mann Whitney non-parametric test. Statistical significance was defined as *p* < 0.05. Survival data was analysed with the Kaplan-Meier method and the log-rank (Mantel-Cox) test.

### Ethical approval

All mouse studies were approved by the animal ethics committee of the LUMC. Experiments were performed in accordance with the Dutch Act on Animal Experimentation and EU Directive 2010/63/EU (‘On the protection of animals used for scientific purposes’).

## Results

### Anti-neu mAb therapy improved survival in transgenic BALB/c-NeuT spontaneous tumour model

**Figure 1:**
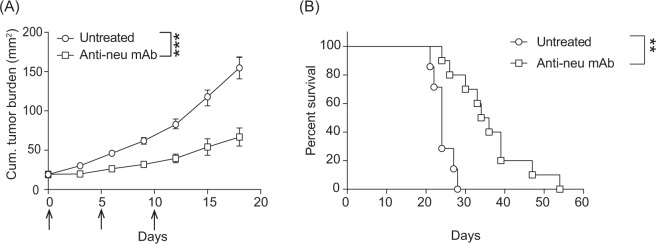
Effective anti-tumour response of anti-neu mAb therapy against spontaneous mammary carcinomas. (**A**) When cumulative tumours reached an average size of 20 mm^2^, NeuT female mice were randomly distributed to the treated and untreated groups. The treatment group was subsequently injected intraperitoneally with 100 µg anti-neu (Day 0, 5, and 10; arrows). Mean tumour size ± SEM is shown. Statistical significance was determined by Mann Whitney non-parametric test (*P < 0.05; **P < 0.01; ***P < 0.001). (**B**) Data from A presented as Kaplan-Meier survival curves. Logrank test was used to determine the statistical significance of the survival. Pooled data of six independent experiments, 7 to 12 animals per group.

We first analysed the anti-tumour effect of anti-neu mAb monotherapy in the BABL/c-NeuT female mice, which develop spontaneously invasive mammary tumours around 4 months of age^13,15,21^. As shown in Fig. 1A, administration of anti-neu mAb to spontaneous mammary tumours bearing BALB/c-NeuT mice resulted in significant delay in tumour outgrowth (p < 0.001), leading to significant improvement in survival when compared with untreated mice (Fig. 1B) (p < 0.01). Our results support previous studies showing the therapeutic effect of anti-neu antibodies *in vivo*^8,14^.

### Sub-optimal TUBO tumour take in WT BALB/c recipient mice

Similar to the majority of the oncogene-driven models of cancer in genetically engineered mice (GEMMs)^22,23^, spontaneous mammary tumours driven by activated rat-neu in the MMTV-NeuT mouse model may not harbour high neo-antigen load^24^. To evaluate whether the therapeutic efficacy of anti-neu mAb therapy would be stronger when the tumour is more immunogenic, we used a transplantable tumour model, the oncogenic rat-neu (NeuT) expressing tumour cell line (TUBO) established from a spontaneously developed mammary gland tumour of a female BALB/c-NeuT mouse^12^. As recipients of the tumour cells, we used either non-syngeneic WT BALB/c mice in which, due to the fact that the rat-neu molecule is a non-self-antigen, the tumour might be highly immunogenic, or syngeneic transgenic BALB/c-NeuT mice for which the rat-neu is a self-antigen and therefore the tumour might be poorly immunogenic^25^.

**Figure 2:**
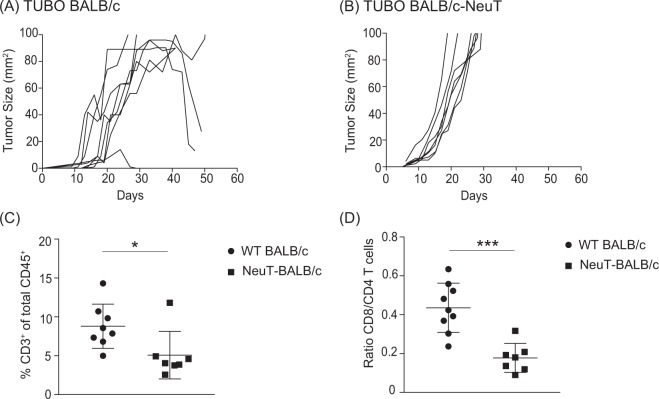
Inconsistent growth of established TUBO tumour in WT BALB/c mice. WT (**A**) and BALB/c-NeuT (**B**) mice were injected subcutaneously with 5 × 10^5^ TUBO tumour cells. Individual tumour growth curves are shown. Tumour cells were injected into WT or BALB/c-NeuT mice, established tumours were harvested on day 15 and analysed with flow cytometry for the percentages of the total CD3^+^ T cells (**C**) or the ratio of CD8/CD4^+^ T cells (**D**), Data are represented as mean ± SEM. Statistical significance was determined by Mann Whitney non-parametric test (*P < 0.05; **P < 0.01; ***P < 0.001). Pooled data of two independent experiments, 7 to 8 animals per group.

WT BALB/c and transgenic BALB/c-NeuT female mice were injected subcutaneously with TUBO tumour cells and tumour outgrowth was monitored. All WT BALB/c mice had palpable tumours at around day 8 upon transplantation, however, the growth of established tumours was inconsistent, and some underwent spontaneous regression without therapeutic intervention (Fig. 2A). In contrast, spontaneous regression of established TUBO tumours did not occur in syngeneic BALB/c-NeuT mice (Fig. 2B). These observations suggest that TUBO cells are more immunogenic in WT BALB/c than in BALB/c-NeuT recipient mice. Flow cytometric analysis revealed that there was an increase in percentage of total CD3^+^ T cells and in CD8^+^/CD4^+^ T cell ratio in TUBO tumours from WT BALB/c mice compared to TUBO tumours from BALB/c-NeuT mice (Fig. 2C,D), suggesting that T cells may contribute to the tumour regression in WT BALB/c mice.

### Stronger therapeutic efficacy of Anti-neu mAb in TUBO bearing WT than BALB/c-NeuT mice

**Figure 3:**
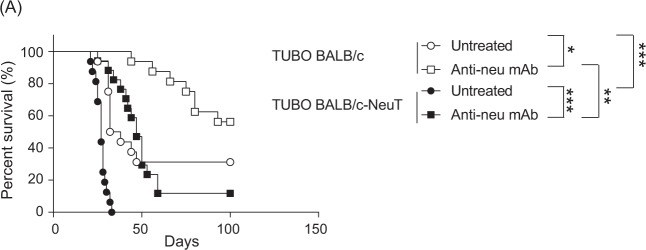
Stronger therapeutic efficacy of Anti-neu mAb in TUBO bearing WT than BALB/c-NeuT mice. (**A**) WT BALB/c and BALB/c-NeuT female mice were injected subcutaneously with 5 × 10^5^ TUBO cells and treated intraperitoneally with 100 µg anti-neu (Day 10, 15, and 20). Data is presented as Kaplan-Meier survival curves. The mice were followed for tumor outgrowth until day 100 and were sacrificed when the tumour reached 100 mm^2^. Logrank test was used to determine the statistical significance of the survival (*P < 0.05; **P < 0.01; ***P < 0.001). Pooled data of two independent experiments, 16 to 17 animals per group.

Based on the difference in immunogenicity of TUBO in WT and BALB/c-NeuT mice, we anticipated different therapeutic responses to anti-neu mAb therapy in WT and BALB/c-NeuT mice. There was a significant increase in survival of untreated TUBO bearing WT mice compared to untreated TUBO bearing BALB/c-NeuT mice, in keeping with the earlier suggestion that an effective endogenous anti-tumour immune responses against the TUBO tumour is induced in WT but not BALB/c-NeuT mice (Fig. 3A). Short-term treatment with anti-neu mAb significantly increased the survival of both TUBO bearing WT and BALB/c-NeuT mice. However, the survival of WT BALB/c was significantly higher compared to the survival of BALB/c-NeuT mice. Taken together, our data strongly suggest that the response of established TUBO tumours to anti-neu mAb treatment correlates with the immunogenicity of tumours.

### Anti-neu mAb monotherapy enhances the anti-tumour T cell responses against immunogenic tumours

**Figure 4:**
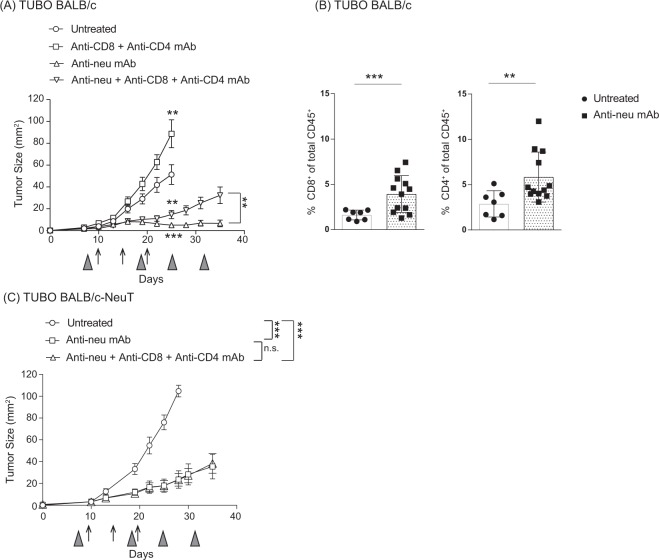
Anti-neu mAb monotherapy enhances the anti-tumour T cell responses to further delay the outgrowth of immunogenic tumours. (**A**) WT BALB/c female mice were injected subcutaneously with 5 × 10^5^ TUBO cells and treated intraperitoneally with 100 µg anti-neu (Day 10, 15, and 20; arrows) and/or 100 µg anti-CD8 (Day 8, Day 18 and weekly; grey triangles) and anti-CD4 mAb (GK1.5; Day 8, Day 18 and weekly; grey triangles). Data from A presented as average tumour outgrowth (mm^2^) ± SEM. Statistical significance was determined by Mann-Whitney test (*P < 0.05; **P < 0.01; ***P < 0.001). (**B**) Tumour bearing WT BALB/c mice were treated with anti-neu mAb on Day 10 post tumour inoculation, tumours were resected on Day 15 and analysed for the presence of CD4^+^ and CD8^+^ T cells via flow cytometry. Statistical significance was determined by Mann-Whitney test (*P < 0.05; **P < 0.01; ***P < 0.001). Pooled data of three independent experiments. (**C**) Same as A except BALB/c-NeuT mice were used. Pooled data of two independent experiments, 6 to 9 animals per group.

To investigate the role of T cells in the anti-tumour activity of anti-neu mAb in both WT and BALB/c-NeuT mice, this therapy was tested in mice depleted for CD4^+^ and CD8^+^ T cells. Efficiency of T cell depletion was validated during the experiment (Supplemental Fig. 1A,B). As expected, depletion of T cells clearly increased the growth of established TUBO tumours in WT BALB/c mice (Fig. 4A). Whereas treatment with anti-neu mAb resulted in strong tumour regression in WT BALB/c mice, tumours relapsed progressively in the absence of T cells, suggesting that T cells are essential for the maximal therapeutic efficacy of anti-neu mAb. In a follow-up experiment, we observed increased CD4^+^ and CD8^+^ T cells in the TUBO tumour of WT BALB/c mice treated with anti-neu mAb (Fig. 4B). This increase was not observed in BALB/c-NeuT mice (data not shown). Together, these data indicate that in immunogenic tumours, anti-neu mAb therapy contributes to strong tumour growth inhibition and eradication by improving the anti-tumour T cell responses.

Markedly delayed outgrowth of TUBO tumours was also observed in BALB/c-NeuT mice (Fig. 4C). However, the therapeutic efficacy of anti-neu mAb was not impaired in the absence of CD4^+^ and CD8^+^ T cells (Fig. 4C), indicating that the T cells in BALB/c-NeuT mice did not contribute to the therapy in contrast to the T cells in WT BALB/c mice. Together, our results suggest that T cells are responsible for the poor TUBO tumour outgrowth in WT BALB/c mice and that anti-neu mAb monotherapy can enhance the anti-tumour T cell responses to further delay the tumour outgrowth. In contrast, the much weaker anti-neu mAb-mediated anti-tumour effect in TUBO bearing BALB/c-NeuT mice, in which the tumour is less immunogenic, is T cells independent. When T cells were absent in WT BALB/c mice, the anti-tumour response of anti-neu mAb resembled strongly the response in BALB/c-NeuT mice, indicating a T cell-independent tumour-outgrowth inhibition by anti-neu mAb.

## Discussion

Here we report that the immunogenicity of mouse mammary tumours can have a significant impact on the response to anti-neu mAb immunotherapy. When rat-neu is a non-self-antigen, making the rat-neu^+^ tumour highly immunogenic as in WT BALB/c recipient mice, T cells contributed to a more pronounced anti-neu mAb-mediated tumour growth inhibition and eradication of the transplanted tumours than when rat-neu on the tumour is a self-antigen, as in BALB/c-NeuT recipient mice. In the latter mice, the weaker anti-tumour response induced by anti-neu mAb therapy was T cell independent.

Our observation that rat-neu expressing TUBO tumours are highly immunogenic in WT BALB/c mice is in agreement with the finding of Reilly *et al*.^26^ who demonstrated that the minimum tumour cell dose required for tumour outgrowth in 100% of the transplanted animals was 100-fold lower for the neu transgenic mice compared with non-transgenic mice. Our study also confirms previous studies showing that CD4^+^ and CD8^+^ T cells can contribute to anti-neu mAb monotherapy to suppress the growth of the TUBO tumour in WT BALB/c immunocompetent mice^8,14^. However, we did not observe a contribution of T cells to the therapeutic efficacy in anti-neu mAb treated TUBO bearing BALB/c-NeuT transgenic mice. An important question raising from these experiments is: why T cells contributed to the therapeutic efficacy of anti-neu mAb in WT BALB/c but not BALB/c-NeuT tumour bearing mice? A simple explanation is that rat-neu is a foreign antigen in normal BALB/c mice, and that the rejection of the TUBO tumour likely involved high avidity rat-neu specific T cells that are strongly, and rapidly activated and expanded after transplantation. This hypothesis is in agreement with our data showing the poor TUBO tumour growth and development in WT BALB/c mice. In contrast, BALB/c-NeuT mice which express rat-neu as self-antigen, harbour lower numbers of self-reactive low-avidity T cells which are more difficult to activate and do not expand effectively^27–29^. It has been demonstrated that spontaneous Her2-driven mammary tumorigenesis in BALB/c-NeuT animals is not suppressed by the adaptive immune system^15^, suggesting the lack of neo-antigens that can be recognised by T cells.

By depleting CD8^+^ T cells, Park *et al*.^8^ and Stagg *et al*.^11^ also noted the importance of CD8^+^ T cells in anti-neu mAb therapy in TUBO tumor bearing NeuT transgenic animals resulting in complete tumor regression in 20% of the mice. This is not in agreement with our results and it might be due to differences in experimental conditions. Park *et al*.^8^ used F1 rat-neu transgenic mice (BALB/c x FVB/N MMTV-neu) as the recipient of TUBO cells. Although these mice are ‘tolerized’ for rat neu, most likely there are still genetic differences between the TUBO cell line and the F1 BALB/c x FVB/N MMTV-neu recipient mouse, which can explain the stronger, partially CD8^+^ T cell dependent, anti-TUBO immune response they report, compared to our studies in the fully syngenic BALB/c-NeuT mice. Besides, all other experiments of Park *et al*.^8^ showing dependence on adaptive and innate immunity of anti-neu mAb therapy were performed in WT BALB/c or KO BALB/c mice not tolerized for the rat-neu antigen^11^. By using other rat-neu^+^ tumor cell lines (H2N100, H2N113, and H2N67) derived from the same BALB/c-NeuT mice as the TUBO cell line, transplanted onto full syngenic BALB/c-NeuT recipient mice Stagg *et al*. confirmed the essential role of CD8^+^ T cells in anti-neu mAb therapy. However, in contrast to Park *et al*. they observed only a moderate delay in tumor outgrowth and no complete regression in a subset of treated mice., Side by side *in vivo* comparison of the therapeutic efficacy of anti-neu mAb in both the H2N and the TUBO model would be necessary to exclude that differences in immunogenicity between these tumor cell lines underlie the different outcome of the anti-neu mAb therapy experiments of Stagg *et al*. and the experiments described here. Furthermore, Stagg *et al*.^11^ used a much more intensive treatment regime (8 times 100 µg anti-neu Ab within a period of 14 days) than we did (3 times 100 µg anti-neu Ab within 10 days). It could be that sustained tumour cell killing by a longer period of treatment leads to induction of a strong inflammatory response activating low avidity anti-neu CD8^+^ T cells. This might also explain the syngergistic effect of anti-neu mAb and anti-PD-1 mAb^11^ in this setting. Notably, CD8^+^ T cell dependency has also been reported for the effective combination therapy of anti-death receptor 5 (DR5) and anti-neu mAb in BALB/c-NeuT mice^30^. Since anti-DR-5 mAb triggers mainly apoptosis in tumour cells, this study raises the possibility that the sustained release of danger signals or damage-associated molecular patterns (DAMPs) from tumour cell death could be key at breaking immune tolerance and inducing meaningful CD8^+^ T cell antitumor immunity in neu-transgenic mice. This might as well explain why neu-specific whole tumour vaccination^26^ but not DNA vaccination^12,31,32^ therapies elicit antitumor CD8^+^ T cell responses in neu transgenic mice.

 There is an increasing amount of literature supporting that anti-tumour immunity of anti-neu mAb can be enhanced with immunomodulatory agents such as CD73^33^, PolyI:C and CpG^34^ in the highly immunogenic TUBO transplanted WT BALB/c mice. Our study suggests that the potential and underlying mechanisms of action of these combination therapies have to be elucidated in low immunogenic setting using transgenic BALB/c-NeuT recipient mice in order to complement the results obtained in WT BALB/c mice. Our result in BALB/c-NeuT mice suggest that direct tumour growth inhibition by blockade of rat-neu signalling is one of the major mechanisms of anti-neu mAb in transgenic NeuT mice. Improved anti-tumour effects could be achieved by the continuation of treatment of these mice with anti-neu mAb, similar to the therapy in breast cancer patients who often receive anti-Her2 mAb over long periods of time^35^ or combining it with a tumour targeting agent such as anti-DR5 mAb that induces tumour cell death directly^11^. We do not rule out, however, that Fc-mediated effector functions might be involved also in anti-neu mAb therapy. Further investigations are needed to reveal the contribution of FcγR and FcγR expressing immune cells to the anti-tumour efficacy of anti-neu mAb and whether FcγR expression can be modulated in the tumour microenvironment to augment mAb-mediated^36^ effector functions for both immunogenic and non-immunogenic tumours. Our study supports the notion that intense immune phenotyping of the various syngeneic tumour models is critical for both rational model selection and data interpretation for clinical translation^37–39^. For example, the high immunogenicity of a fully non-self-antigen such as rat-neu in the TUBO tumour bearing WT BALB/c model, will most likely result in overestimation of the potency of combining anti-neu mAb therapy with immunotherapy. On the other hand, using a completely syngeneic model such as MMTV BALB/c-NeuT mice that develop spontaneous mammary tumours or TUBO transplanted in fully syngeneic BALB/c-NeuT mice might result in underestimation of the potential anti-tumour immune response in patients, in which mutations may have led to neo-epitope formation and a certain level of immunogenicity.

Antibody targeting Her2 (trastuzumab, pertuzumab) is among the first of the approved antibodies directed against Her2^+^ cancer cells. Although these antibodies have become standard of care and improved overall survival of patients with Her2^+^ breast cancer, heterogeneity exists within Her2-positive tumours, and the overall response rate to anti-Her2 mAb-based therapies remains modest, approximately 26% when used as a single therapy and 40–60% when used in combination with chemotherapy^2,40,41^. To date, Her2 expression level remains the only suitable marker for patient selection for anti-Her2 based regimen^42,43^. Nonetheless, the relationship between the level of Her2 amplification and efficacy of trastuzumab based therapy in adjuvant treatment remains controversial^44^. Two studies^45,46^ showed no correlation and one showed a negative correlation^47^ between Her2 amplification and clinical survival. Clearly, beyond Her2 testing, there is a need to identify more reliable biomarkers to better predict which patients respond to this treatment. Unlike melanoma and lung cancer, the majority of breast cancer has not been considered immunogenic owing to its relatively low mutational load and hence low repertoire of neo-antigens^48,49^. These poorly immunogenic breast tumours are less likely to benefit from cancer immunotherapy. Nonetheless, high mutational burden^50^ and an increase of tumour infiltrating lymphocytes (TILs)^51–54^ can be found in some Her2^+^ breast cancer patients. Several studies have shown that TILs are significantly associated with improved survival in Her2^+^ breast cancer, as well as better response to anti-her2 mAb therapy^52–55^. These observations suggest that host immunity can contribute to the antitumour activity of Her2-targeted agents. Here, we compared the impact of the difference in immunogenicity of the TUBO tumour in non-isogenic WT and isogenic transgenic BALB/c-NeuT mice on the therapeutic efficacy of anti-neu mAb therapy. We observed an increased infiltration of T cells into the untreated established TUBO tumours from WT BALB/c mice compared to untreated tumours in transgenic BALB/c-NeuT mice. The higher immunogenicity of the tumours in WT BALB/c mice correlated with increased efficacy of anti-neu mAb therapy compared to the therapeutic efficacy in BALB/c-NeuT mice. Taken together, our study suggests that selection of HER2^+^ breast cancer patients based on the level of pre-existing TILs may be useful and critical for an optimal anti-Her2 mAb-based combination therapy with better therapeutic outcome.

## Supplementary information

Supplementary Information
