# Supplementary material for: Immunogenicity of rat-neu+ mouse mammary tumours determines the T cell-dependent therapeutic efficacy of anti-neu monoclonal antibody treatment
Source: Sci Rep. 2020 Mar 3;10:3933. doi: 10.1038/s41598-020-60893-8 (PMC7054273; doi:10.1038/s41598-020-60893-8)
Supplement: Supplementary file 1 — Supplementary Information [file 41598_2020_60893_MOESM1_ESM.docx]

**Immunogenicity of rat-neu^+^ mouse mammary tumours determines the T cell-dependent therapeutic efficacy of anti-neu monoclonal antibody treatment**

Heng Sheng Sow^1^, Hreinn Benonisson^1^, Margot M. Linssen^1^, Conny Brouwers^1^, Marcel Camps^2^, Cor Breukel^1^, Jill Claassens^1^, Thorbald van Hall^3^, Ferry Ossendorp^2^, Marieke F. Fransen^2*^, and J. Sjef Verbeek^1*&^

1. Department of Human Genetics, Leiden University Medical Centre, Leiden, the Netherlands

2. Department of Immunohematology and Blood Transfusion, Leiden University Medical Centre, Leiden, the Netherlands

3. Department of Medical Oncology, Leiden University Medical Centre, Leiden, the Netherlands

*Equally contributed last author

^&^ Corresponding author

# current address: Department of Pulmonary Diseases, Amsterdam University Medical Centre, AmsterdM, The Netherlands. m.f.fransen@amsterdamumc.nl


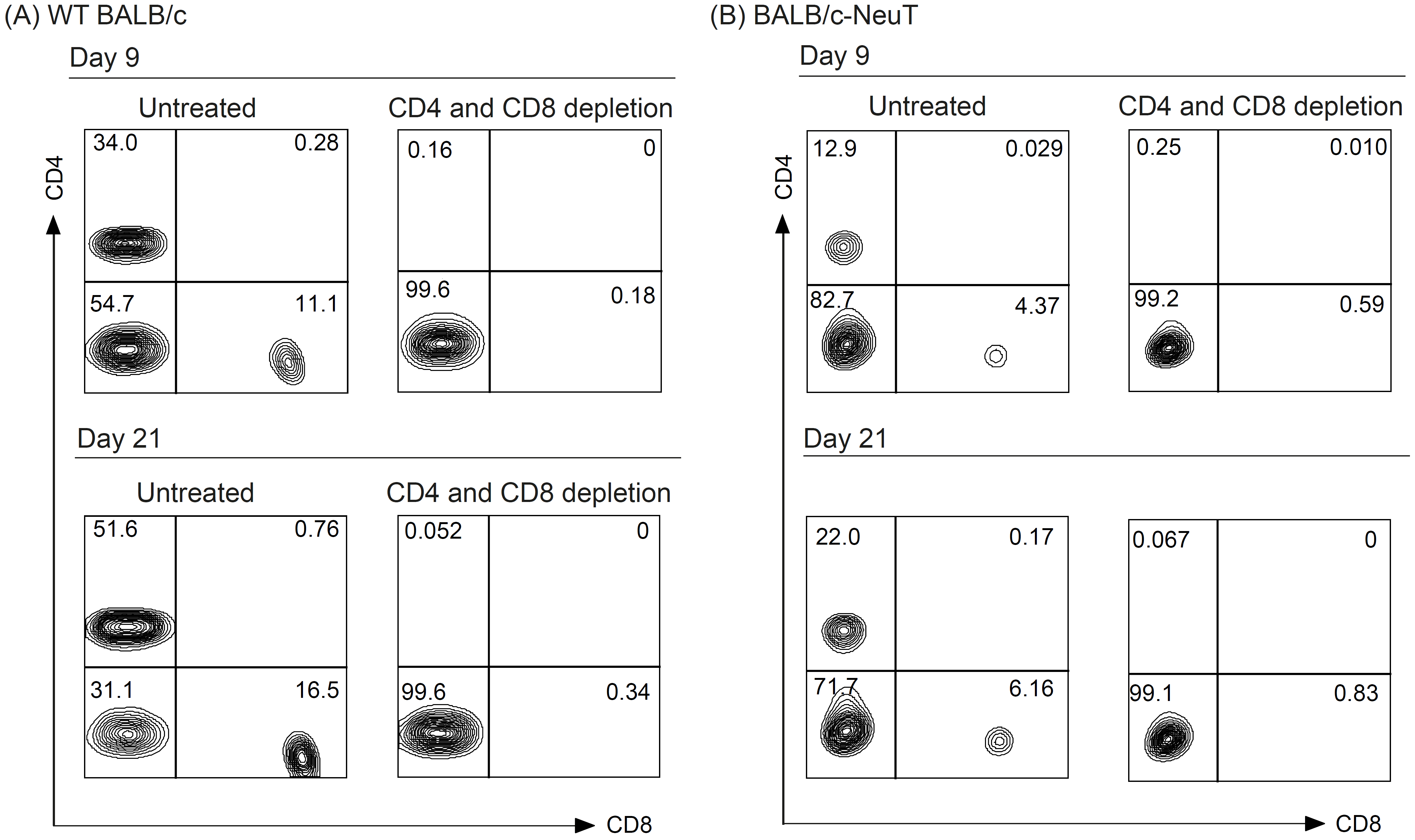


**Supplementary Figure 1. Conformation of *in vivo* depletion efficacy.** Facs dot plots show the frequencies of CD4 and CD8 T cells in blood of WT BALB/c (A) and BALB/c-NeuT (B) at Day 9 and Day 21 which received CD4 and CD8 depleting antibodies at Day 8 and 18.
